# Regarding: Regional variation in low-value musculoskeletal surgery: a nationwide study from the Finnish Care Register

**DOI:** 10.2340/17453674.2024.42631

**Published:** 2024-12-23

**Authors:** Christopher W DIGIOVANNI, C Niek VAN DIJK, Mark GLAZEBROOK, Helka KOIVU, Mikko HAUTAMÄKI, Anssi HÄRKÖNEN, Masato TAKAO, Manfred THOMAS, Marko MYKKÄNEN, James W STONE, Alastair YOUNGER

**Affiliations:** 1 Department of Orthopaedic Surgery, Massachusetts General and Newton-Wellesley Hospitals, Harvard Medical School, Boston, MA, USA; 2 University of Amsterdam, Department of Orthopedic Surgery, Amsterdam UMC location AMC, the Netherlands; 3 Dalhousie University, Reconstructive Foot & Ankle Surgery and Orthopedic Sports Medicine, Queen Elizabeth II Health Sciences Center Halifax Infirmary, Halifax, Nova Scotia, Canada; 4 Hospital Pihlajalinna Turku Kupittaa, University of Turku, Turku, Finland; 5 Hospital Pihlajalinna Helsinki, Helsinki, Finland; 6 Kuopio University Hospital, Kuopio, Finland; 7 Jujo Hospital, Mangoku, Kisarazu, Chiba, Japan; 8 Hospital, Augsburg, Germany; 9 Orthopedic Institute of Wisconsin, Milwaukee Wisconsin, USA; 10 Department of Orthopaedics, University of British Columbia, Canada

*Sir*,—We read with interest the study “Regional variation in low-value musculoskeletal surgery: a nationwide study from the Finnish Care Register” by Ponkilainen et al., 2024 [1]. We would like to comment on the methodology and the authors’ conclusions regarding ankle arthroscopy.

The authors classified ankle arthroscopy as a “low-value surgical procedure” based on an outdated 2009 review by Glazebrook et al. [2]. However, this review’s conclusions were misinterpreted. 3 indications for ankle arthroscopy received a grade B recommendation, meaning they were supported by good evidence from Level II or III studies [3]. Few foot and ankle procedures achieve this level of support. While some indications had grade I (incomplete) or C (limited support) recommendations, only 1 (ankle arthritis debridement) argued against using ankle arthroscopy.

The lack of RCTs is mainly due to the clear benefits of minimally invasive surgery, with the non-inferiority of open surgery justifying the adoption of arthroscopic approaches. Methods used in drug research are not suitable for surgical outcomes. Since the introduction of arthroscopy, many open procedures have been replaced by arthroscopic ones with minimal RCTs. For example, an RCT has never been conducted comparing open meniscectomy with arthroscopic meniscectomy. Systematic reviews consistently show advantages and better outcomes for various joints, including the knee and shoulder. Similarly, endoscopic ankle surgery has advanced over the last 25 years, reducing complications and enabling faster recovery.

Operative treatment for anterior and posterior ankle impingement, lateral ankle instability, and ankle arthrodesis has a long history. Recent systematic reviews have demonstrated that an arthroscopic approach is at least non-inferior to open surgery. A systematic review of arthroscopic treatment for anterior ankle impingement found an 81% success rate [4]. The advantages of arthroscopy are so clear that an RCT comparing open versus arthroscopic treatment for this condition is unlikely ever to be conducted. Arthroscopic bone marrow stimulation (BMS) is a minimally invasive, cost-effective procedure with low complication rates and satisfactory results [5-7]. Meta-analyses also show that arthroscopic repair of chronic lateral instability results in better outcomes and fewer complications than open repair [8-10]. Arthroscopic ankle arthrodesis has shown higher fusion rates, less blood loss, and shorter surgery times than open arthrodesis [11-12]. Similarly, endoscopic treatment for posterior ankle impingement has significantly fewer complications than open surgery [13]. Endoscopic calcaneoplasty for Haglund’s deformity provides better functional outcomes, fewer failures, and faster recovery than open surgery [14].

The reliability of the Finnish Care Register for Health Care (Hilmo) can also be questioned. In practice, many have found procedure codes false when conducting register studies, possibly due to often vague and unsuitable NOMESCO codes. The Hilmo data should be validated before being used to draw restrictive conclusions about specific procedures.

## Conclusion

We find it disturbing that the journal’s peer-review process would allow the publication of such poor-quality scientific methodologies. This may mislead readers into thinking ankle arthroscopy should not be performed, potentially harming this valuable procedure and hindering ongoing research on evolving techniques that continue to demonstrate positive outcomes.
